# AIDS Inside and Out: HIV/AIDS and Penal Policy in Ireland and England & Wales in the 1980s and 1990s

**DOI:** 10.1093/shm/hky090

**Published:** 2018-10-25

**Authors:** Janet Weston, Virginia Berridge

**Affiliations:** * Centre for History in Public Health, London School of Hygiene & Tropical Medicine, 15–17 Tavistock Place, London WC1H 9SH, UK

**Keywords:** HIV/AIDS, policy, prisons, Ireland, England and Wales

## Abstract

As HIV/AIDS emerged in the 1980s as a new and seemingly overwhelming public health challenge, prisons were highlighted as an important location for the control of the epidemic. Yet, they often seemed unwilling or unable to adopt national guidelines. This article compares the policy decisions made by the prison services of the Republic of Ireland and England & Wales in response to HIV/AIDS in the 1980s and 1990s, bringing together the histories of penal policy and HIV/AIDS for the first time. It develops our understanding of contemporary policy history, and demonstrates the value of a comparative approach to both penal and health histories. Policy-making was shaped by both national and more localised traditions and trends, from attitudes to criminal justice and responses to HIV/AIDS at the national level, to the histories, structures, and staffing of prison services themselves.

As HIV/AIDS emerged in the 1980s as a new and seemingly overwhelming public health challenge, prisons were singled out as a likely flash point for its spread and an important location for controlling the potential epidemic.^1^ The large numbers of injecting drug users passing through prisons were perceived as a hard-to-reach population at risk of HIV/AIDS, and a dangerous ‘bridge’ between at-risk minority groups and the heterosexual community. Prisons themselves were also seen as sites of contagion, where sex, drug use, violence and squalor might all conceivably contribute to the spread of HIV/AIDS behind, and then beyond, prison walls.^2^ International bodies developed policies and guidance dedicated to this new and potentially devastating problem, and called for prisons to shoulder their public health responsibilities.^3^

Policy making of this kind was a frequent focus of initial research into interpretations of HIV/AIDS.^4^ Often seen were two broad strands of argument. One situated HIV/AIDS policy as part of the reaction of the New Right, epitomised by the Thatcher government of the 1980s. This was marked by the rhetoric of the mass media with its talk of a ‘gay plague’, and an upsurge of moralising about ‘innocent’ and ‘guilty’ victims of the epidemic.^5^ New Right governments were engaged in a crusade against the excesses of the 1960s and 1970s by this point, and their approach to HIV/AIDS was characterised as one of moral panic. An alternative analysis came from policy scientists and historians. The former argued that the populist backlash had little effect on the actual nature of government policy making, since policy responses were primarily shaped by the technical doctrines and liberal values of the traditional biomedical elite, by the ‘power of professionalism’.^6^ Historians, including the co-author of this article, accepted this approach but pointed to change in policy influence over time. The influence of newcomers to the policy sphere, such as groups from the gay community, also carried considerable weight, especially in the early days of the epidemic. The clinical specialities involved, such as genitourinary medicine, were not usually high profile in policy terms but gained considerable influence by way of HIV/AIDS.^7^

Historians also pointed to the importance of ‘pre-history’. Policy making did not occur in a vacuum, but was clearly influenced by the traditions of particular policy areas and by pre-existing issues and tensions.^8^ A broadly ‘liberal consensus’ surrounding HIV/AIDS policy was further bolstered by historical comparisons, which highlighted the failures of the past in trying to deal with epidemics or STDs by restrictive and punitive measures.^9^ Prisons, however, appeared resistant to the charms of public health, activism, and history. Prisons across Europe and beyond were criticised for an array of failings, the most contentious and long-standing of which were the segregation of prisoners with HIV/AIDS and a reluctance to acknowledge risky sex and drug taking behind prison walls.^10^ Prisons did not seem to fit the idea of a ‘liberal consensus’.

A comparative analysis of HIV/AIDS policy-making for prisons in the 1980s and 1990s forms the focus of this article, which contrasts the Irish Prison Service and Her Majesty’s Prison Service of England & Wales (HMPS).^11^ Although both services were criticised, their policies differed in important respects. In fact, HMPS policy bore a closer relationship to the ‘liberal consensus’ than has perhaps been recognised—closer, certainly, than was the case in the Republic of Ireland in the 1980s and early 1990s. However, in the later 1990s, it was Irish policy that underwent the most dramatic change. We argue that these differences reflect the national contexts and prison systems within which HIV/AIDS policy was formulated. These distinct contexts into which HIV/AIDS emerged are described in more detail in the first section of this article, and we return to them throughout.

Our analysis considers initial reactions to discoveries of HIV within prisons in both jurisdictions, following which segregation loomed large, before moving on to examine the 1990s and long-running issues of condom provision and addiction treatment. The comparison of the Republic of Ireland with England & Wales reveals that four interconnected factors were of particular importance in shaping HIV/AIDS policy. First, the histories and structures of these two prison systems, including the legacy of political prisoners and the extent of external scrutiny, were significant. Second is the level and nature of political attention granted to prisons and punishment. This changed for both jurisdictions in the 1990s, albeit in different ways, but in both cases one result was a less insular prison system which brought penal policy closer into line with national standards. Thirdly, the presence or absence of medical professionals within prisons and within policy making structures was key. This remained the case into the 1990s and beyond, although as the example of HMPS shows, a greater medical presence was not always to the benefit of policy implementation. Lastly, broader national responses to HIV/AIDS were influential, for both good and ill. What therefore characterised penal policy making in both jurisdictions is the weight of institutional pressures and structures, as well as the broader social and political context.

There is a current revival of HIV/AIDS as a historical subject, but this has shown less interest to date in the policy analysis which marked the earlier historiography.^12^ There are signs that this is beginning to change, particularly for the history of the United States (USA) but across Europe as well.^13^ US prisons have also attracted greater attention from historians thanks to the centrality of mass incarceration to the modern American landscape. In responding to Emily K. Hobson’s recent call for greater attention to the overlapping histories of AIDS and prisons, we shift the focus away from the USA, add nuance to orthodox interpretations of policy making and contribute to growing interest in prisons as sites of health care and health policy.^14^

This article deals with very recent history, but the methodology of government archives, grey literature, official reports and news items is familiar to those who work on the history of policy. In one area, the use of oral history, some further comment is needed. This article draws upon interviews with 30 civil servants, prison staff, voluntary sector workers, probation officers, clinicians and former prisoners from both England and Ireland, conducted in 2016–17. It has also made use of earlier interviews, anonymised at the time, which were conducted by Virginia Berridge in the late 1980s and early 1990s for a study of HIV/AIDS policy making in the UK. Many of these informants have since died, including Dr Kilgour, then Director of the Prison Medical Service, and Len Curran, HMPS psychologist who played a leading role in the difficult task of developing research on HIV/AIDS in prisons in England & Wales.^15^

This has prompted reflection on the differences between researching HIV/AIDS policy making in the 1990s and in 2017. The recent revival of historical interest in HIV/AIDS reflects its redefinition as ‘real history’, whereas before it was sometimes viewed as ‘slow journalism’. In the 1990s, archives were more difficult to come by, often handed over ‘under the counter’.^16^ Interviewees were in the thick of events, sometimes very open in their discussion of current problems but anxious not to be directly quoted. Kilgour, for example, was frank in his interview about his use of international networks through the World Health Organisation (WHO), as well as UK networks, to try to force change within HMPS and to establish a liberal response. The enormous fear of AIDS also comes over very clearly. Now, by contrast, the time of fear is gone but informants are much more willing to be quoted and interviewed.^17^ This willingness is particularly marked in the context of the Republic of Ireland, where the history of HIV/AIDS has been subject to relatively little research and many stories are only now being told for the first time. Archival material in the UK is slightly more readily available three decades on from the crisis, although official files remain closed in both jurisdictions and much is still unlisted and undeposited, emerging from attics and office clear-outs only when the historian’s interest in such papers is made clear. Plenty of material has already been lost to the rubbish tip or to the vagaries of early computing software. Those engaged in researching histories of HIV/AIDS should do their best to gather and deposit archives and interviews while the opportunities remain, so that the field can flourish in the future.

## National and Penal Contexts

The 1980s and particularly the 1990s have been seen as transformative decades for the Republic of Ireland. In the early 1980s, Ireland appeared to be an anomaly amongst developed Western nations thanks to its economic failings, its repressive sexual politics and ‘clerical interventionism’, and its religious and territorial conflicts. Paralleling patterns elsewhere, the 1960s and 1970s had been a period of modest but significant social change but were followed by a backlash in the 1980s, stimulated by the visit to Ireland of Pope John Paul II in 1979.^18^ Yet, by the early twenty-first century, as one Irish studies scholar has reflected, ‘Ireland is no longer so different’. Spectacular economic growth, rapid social change, and the Good Friday agreement all seemed to bring it into line with its neighbours.^19^ This may overstate Ireland’s former exceptionalism and the speed and depth of subsequent change, but as we will show, the example of HIV/AIDS and penal policy making does point towards the 1990s as a time of significant flux, much more so in the Republic than was the case for England & Wales.^20^

Irish penal history has focused upon the role of prisons in the ‘containment of political disorder’ before the establishment of the Free State and during the Troubles, and the widespread use of alternative institutions such as psychiatric hospitals and Magdalene laundries for holding socially deviant individuals. The former encouraged the isolation and secrecy of the Department of Justice, which retained tight control over all prison-related decisions, and spared little attention for the ‘ordinary’ prisoner. The latter saw exceptionally low numbers of people in the prisons of the Republic of Ireland throughout much of the twentieth century: well below a thousand in the 1960s and barely 2,000 by the early 1980s. The result, social scientists have argued, was stagnation in policy terms and a widespread lack of interest in prisons overall.^21^ Security was the overwhelming priority. Importantly, there was little involvement in prison administration beyond the Minister, a small cadre of civil servants within the Department of Justice, and the Governors and other staff on the ground. There were no medical professionals in management or advisory positions by the 1980s, no nurses and very few general medical practitioners employed in prisons, no external inquiries or investigations into prison management, and virtually no input from other sectors. HIV/AIDS was to demand the attention of an isolated group, unaccustomed to considering matters of health. In this, the prison system of the Republic of Ireland differed from its counterpart in England & Wales.

In England & Wales, the prison service was much larger and had been growing since the 1950s. By the 1980s, the Home Office was responsible for over 100 establishments and tens of thousands of inmates, managed through its Prisons Board and reviewed by an independent inspector. Histories of the prison service of England & Wales have identified significant debate and change over the twentieth century, with interest in the rehabilitation of offenders keeping penal questions alive and maintaining the expansion and influence of its medical service.^22^ The Director of Prison Medicine was a member of the Prisons Board, and by the 1980s there was also a Deputy Medical Director and Regional Medical Officers, a Head of Nursing and a Head of Pharmacy, all of whom were closely involved in matters of policy and management oversight, plus nearly 100 full time medical officers and many more part time GPs and visiting specialists.^23^ When enthusiasm for rehabilitation foundered in the 1960s and 1970s, however, both the prison service and its medical branch became the object of growing public scrutiny. Formal enquiries were joined by critical television programmes, new voluntary and advocacy bodies, and considerable academic interest.^24^ Criticism of the medical service highlighted its separation from the National Health Service (NHS) and its perceived affiliation with the disciplinary function of the prison, both of which threw into question the priorities of its staff and the quality of care delivered.^25^

In both jurisdictions, the 1990s have been identified as a point of intensification. Where once political prisoners had occupied the lion’s share of attention, the prisons of the Republic of Ireland began to face the new problems of overcrowding, drug use and violence. This, in combination with much greater public interest in matters of law and order, prompted the stirrings of a new approach to prison administration.^26^ One facet of this was the creation in 1999 of a new agency, the Irish Prison Service, complete with advisory board and, from 2002, an independent Prisons Inspector. This effort to promote professionalism and transparency had the potential to affect health care significantly, thanks to new research, staffing and standards. In England & Wales, pressure on prisons was magnified by the political mobilisation of a ‘tough on crime’ stance from across the political spectrum, with trends for tight security and punitive regimes gathering pace.^27^ This presented some challenges for HIV/AIDS-related policy, as we will show. However, the 1990s also saw the arrival of market-like competition within public services, leading to the buying-in of prison services from external providers. This was followed by definitive moves towards integrating prison health care in England & Wales with the NHS, a drawn-out process finally completed in 2006. In different ways, therefore, both prison services were becoming less isolated, which encouraged policies around HIV/AIDS more into line with national standards.

Amidst this sits the HIV/AIDS crisis. The first diagnoses were made in the early 1980s amongst gay men in both jurisdictions, but national perceptions and responses to the looming epidemic soon diverged. Gay groups, voluntary organisations and specialist clinicians put pressure on the UK government to act. By 1986, a sense of crisis prompted national advertising campaigns, the formation of a special Cabinet Committee and a Social Services Select Committee Enquiry, plus funding for pilot needle exchange schemes and biomedical research, quickly followed by the creation of the National AIDS Trust to coordinate the rapidly expanding voluntary sector. Anxiety cooled in the later 1980s as treatments emerged and the numbers of those affected failed to fulfil the worst predictions, and by the 1990s challenges to the liberal consensus were emerging. In the Republic of Ireland, the activities of activist and voluntary groups were more muted and fragmentary. Work on HIV/AIDS in Ireland has highlighted the particularity of Ireland’s conservative political and cultural landscape and the impact of this upon sexual mores and laws *and* state provision for health and social care. Gay Health Action was formed in 1985 and worked to raise awareness and to share information, but as homosexual acts remained illegal until 1993, funding and working relationships with policy makers were all but impossible to obtain.^28^ Extremely divisive national debates in recent years over abortion and divorce left politicians with little appetite for courting controversy, and there was some sense that Ireland was not likely to face a serious epidemic. Responses were slower to emerge and more cautious in tone, with a National AIDS Strategy Committee formed only in 1991. Drug use, rather than homosexuality, was flagged as the most significant route of transmission. These factors inevitably had some impact upon when and how HIV/AIDS in prisons was discussed, and the policies that were pursued.

## 1985/6 and Immediate Decisions: Education and Segregation

Both jurisdictions were spurred into action by the discovery of HIV/AIDS within their prisons. For England & Wales, the death from an AIDS-related illness of the chaplain at Chelmsford prison, Gregory Richards, hit the headlines in early 1985 and led to an element of panic that chimed with the national atmosphere. For a brief period, the Prison Officers’ Association (POA) refused to allow any movement in or out of the prison, bringing home to the prison service the need for an urgent and decisive response to prevent similar episodes elsewhere.^29^ Guidance was immediately issued by the prison medical service management to all prisons on how to recognise and respond to any cases of AIDS, whether amongst staff or inmates, and the Director of Medical Services and his team set out to speak to groups of staff across the country. Dr Rosemary Wool, a Principal Medical Officer at the time and later Director of Medical Services, remembered attending many meetings with a collection of diagrams to try to explain to staff what HIV/AIDS was and how it was transmitted. Dr Pat Lush, the GP for Gloucester Prison, was asked by the Governor there to address staff and inmates together, and the whole prison gathered in the chapel for the purpose. John Ramwell, Hospital Chief Officer at Wakefield prison, recalled hearing the Regional Principal Medical Officer talk through the medical aspects of HIV/AIDS, very much ‘summing up in his own mind how we should deal with it as a society, and particularly in our case in the prison service’.^30^ Prison Medical Director Dr Kilgour, speaking in early 1986, felt that good progress had been made as far as educating staff was concerned, since ‘while we have had threats of industrial action due to, shall we say, over-reaction, due to ignorance of how AIDS is passed’, this danger seemed to have subsided and there had not been any further problems since the initial upheaval at Chelmsford.^31^

HMPS benefited from the knowledge of Dr Kilgour, who had until 1983 worked for the WHO. He made the most of his contacts there in order to stay up to date with the latest information and guidance regarding HIV/AIDS, and to encourage the WHO to produce guidelines on AIDS in prisons that could then be used to advocate for change at home. HMPS also benefited from a large network of medical professionals in senior positions, through which information and policy could be disseminated. At the local level, some prison doctors were then willing and able to share knowledge and reassurances with discipline staff and management teams, although the efficacy of this would depend upon individual doctors and local relationships and structures.^32^ Decision making in the mid-1980s was also informed by the knowledge that scrutiny of the prison medical service was at an all-time high, with a Social Services Committee enquiry on the subject underway and the Prison Reform Trust and editor of the *British Medical Journal* both showing interest. Added to this was the level of media coverage and public anxiety around HIV/AIDS, which was reaching fever pitch in 1985–86.^33^ The actions and inactions of the prison service would not go unnoticed.

One of the first and most significant policy decisions in England & Wales, taken in late 1985, was to apply the Viral Infectivity Regulations (VIR) to prisoners with HIV/AIDS. These regulations had been developed only a short time earlier for hepatitis B, and were to be implemented at the discretion of medical staff. Prison doctors could recommend that prisoners with hepatitis or HIV/AIDS were housed separately from the general population, and excluded from sports activities or work involving sharp objects or food preparation. 34 Official policy regarding the VIR shifted in 1991 in response to criticisms from a variety of sources, including the Prison Reform Trust, whose reports were picked up by the mainstream medical press; Lord Justice Woolf, who had been commissioned to conduct an enquiry into the causes of the 25 day riot at Strangeways prison in 1990; the Advisory Council on the Misuse of Drugs, an influential body set up in the early 1970s to review and advise on policies relating to drugs which produced a number of reports on HIV/AIDS; and voices from within the prison service itself.^35^ Despite these extensive criticisms, revisions to policy were modest, requiring a ‘presumption in favour of “normal” location’ and calling upon medical officers to advise governors of ‘the restrictions considered to be appropriate in the particular case’. This emphasised that the VIR should be an individual and medical decision, not a blanket policy driven by operational concerns.^36^

The discretionary and permissive nature of these policies left considerable scope for interpretation. As with the programme of education that had followed Revd Richards’ death, this approach depended upon the interventions and influence of individual doctors across the prison estate. The relative novelty of the VIR in 1985, in combination with the specific and heightened anxieties about HIV/AIDS at that moment and the reliance upon medical decisions and authority, meant that their implementation was unpredictable. Violence towards prisoners thought to be ‘AIDS carriers’ led to their separation from the general population in some instances, blurring the VIR with operational reasons to segregate inmates at risk.^37^ In some cases, doctors lacked sufficient influence to prevent an element of segregation. At Wandsworth prison, although the senior medical officer refused to house asymptomatic prisoners with HIV/AIDS in the hospital wing, part of the separation wing for vulnerable prisoners was set aside for the same purpose, and remained in use well into the 1990s.^38^ A policy of segregation was also implemented for many years in Liverpool prison, while at other establishments the VIR were never adopted at all.^39^

Analyses of UK prison policy regarding HIV/AIDS have attributed the introduction and variable implementation of the VIR to the isolation of prison medicine from mainstream medicine, and the extent of local independence across the prison estate. These factors allowed prison policy to deviate from national policy by allowing segregation, and made the strict enforcement of blanket policies an alien and challenging proposition.^40^ Indeed, permissive and discretionary policies were always preferred, given that the prison service was responsible for so many different types of prison establishment. The goal of policy, as one senior prison official observed, was to create ‘a framework which tries to set out the boundaries and the parameters’, and then each prison governor and their management team would ‘work it out according to what resources you’ve got, what difficulties you have, and so on’.^41^ These resources and difficulties varied enormously. The quality of relationships between the local POA branch and management, and medical officers and management, also affected local decisions, as did the habits and cultures of particular institutions. In the case of HIV/AIDS, permissive policies which left decisions in the hands of local prison medical officers also reflected the presence of medical expertise throughout the prison service, including in management positions, and an element of confidence in this expertise. As was the case for HIV/AIDS policy on the national stage, more generally, it also indicated a readiness to frame HIV/AIDS as a public health matter for experts to manage, insulating central administration and ministers somewhat from criticism and complaint.

In the Republic of Ireland, the events of 1985 and 1986 took a different path. A burgeoning heroin epidemic in Dublin had prompted an influx of young heroin addicted inmates, and where prisons had stood half-empty only a decade before, there was now increasingly severe overcrowding.^42^ The nation’s largest prison complex, Dublin’s Mountjoy, bore the brunt of this. In October 1985, a handful of injecting drug users in Mountjoy requested an HIV test, and the first positive result prompted something approaching chaos. After one of the visiting GPs had given the prisoner an abrupt diagnosis, word spread rapidly around the prison. Staff brought the prisoner immediately to the administrative area, and later that day, officials in the Department of Justice decided that he should be released. A priest present in the prison at the time remembered that the reaction was to ‘just take a plastic bag, put [the inmate’s belongings] in to it, kick him out’. As the Governor of Mountjoy, John Lonergan, recalled, ‘the hype had started then, the anxiety had started. Within a couple of weeks, I’d say about 20 fellows came back with positive readings’.^43^ The numbers soon increased still further, and a policy of releasing all inmates with HIV/AIDS was clearly unsustainable. Under pressure from the Irish Prison Officers’ Association (IPOA), the Department agreed that prisoners known to have HIV/AIDS would be segregated from the general population. In early 1986, these prisoners were moved to another prison, but after considerable disruption and rioting they were returned to Mountjoy and housed in a separation unit, where some would remain for many years.^44^

As was the case in Chelmsford prison, fears about how easily HIV/AIDS might spread prompted considerable concern amongst staff. But the reaction in policy terms was different, with the Irish prison service immediately introducing a national policy of segregation that was not wholly abandoned for ten years. There were several reasons for this. First, the prison service was following the lessons from its own history. The segregation of political prisoners from the general population of inmates was well-established. Blanket segregation in the name of good order was tried and tested, staff could easily implement it, and the Department of Justice was accustomed to ordering it. Secondly and connectedly, the decision-making process in the Republic relied on a small number of people, and no one with any medical expertise at all. A handful of civil servants and ministers were accustomed to taking all decisions, and in the case of HIV/AIDS this left policy-making susceptible to fears, rumours and pressure from the IPOA. This association was remembered as being ‘very well organised and very powerful, and had huge political influence’: one probation officer recalled that a particular Justice Minister would stop off at the prison officers’ drinking club on a regular basis, and other senior staff felt that ‘across the board, we have to keep the IPOA on side to do anything … prison management were unashamed: that’s our priority’.^45^

In addition to this, expertise and interest in the Republic of Ireland regarding HIV/AIDS, and the activities of prisons, was limited. The state was involved less in matters of public health and social care than was the case in England & Wales, and a fractured and fractious medical profession had little experience in either genito-urinary medicine or addiction, two fields in which HIV/AIDS emerged most urgently. Spending on health care was extremely low compared to other EEC countries, and was facing cut after cut throughout the 1980s.^46^ Activism to elevate the profile of HIV/AIDS was limited, and there had also only been 11 deaths attributed to AIDS by the end of 1985. HIV/AIDS had attracted little attention, and for the prison system it appeared to be a problem confined to Mountjoy that could easily be contained there. In the absence of any likely objections or alternatives, the views of the IPOA and policy-making precedent could hold sway.

A lack of external scrutiny and medical influence helped the policy of segregation to endure, but so, too, did the problems that the policy itself generated or intensified. Distress, mental illness and drug use were not uncommon, especially as time went by and current and former occupants of the separation unit died of AIDS-related illnesses in increasing numbers. Prisoners sometimes cut their arms in protest or desperation, prompting considerable anxiety amongst staff who feared contact with their blood, and in one memorable hostage situation an officer was held with a noose around his neck and threatened with a used syringe. Better facilities in the segregation unit, both perceived and actual, also created problems for reintegration.^47^ Perceptions of those segregated as particularly likely to suffer from severe addiction and mental health problems, and particularly likely both to present a danger to others and to be vulnerable to hostility from other inmates, did little to encourage the idea that they could easily be dispersed amongst the general population. The longer that segregation was enforced, the more difficult it became to dismantle. The same was also true in places like Wandsworth in England & Wales, where elements of segregation proved durable.

Policy for women with HIV/AIDS in Irish prisons changed fairly quickly. Around 1987, a Department of Justice working group advised that segregation should be gradually phased out, and recommended a plan for re-integration that would include staff and prisoner education and the adoption of new guidelines for managing prisoners with HIV/AIDS. These guidelines, with clear echoes of the VIR in England & Wales, stated that inmates known to have HIV/AIDS ‘may be placed on ordinary location in a single cell or accommodation shared with other inmates who are antibody positive’.^48^ This advice was followed for women, and female prisoners who had initially been segregated were returned to the women’s prison. It was adopted for men much more slowly. No reason was ever given for the different treatment of men and women, but it seems likely that sex, violence, hostility towards individuals with HIV/AIDS and the associated risks of HIV transmission and disruptions to security and order, were perceived to be less frequent and less dangerous in the women’s prison.^49^ There was also very little available space for segregated women, with all female prisoners held in a small area of St Patrick’s Institution for juveniles within the Mountjoy complex. For men, by 1993 the Department of Health reported that those known to have HIV/AIDS were no longer being segregated, but prisoners already in the separation unit remained there.^50^ Only after considerable criticism from international bodies, the growing cohort of HIV/AIDS experts in the Republic, the new Director of Prison Medical Services and expert committees, and the construction of a Medical Unit into which some of the more severely addicted and disturbed men with HIV/AIDS could be moved, was the policy of segregation formally abandoned in January 1995.

## The 1990s and Long-Running Concerns: Sex and Drugs

Although policy and practice on segregation took years to resolve, the key policy issues in the 1990s centred upon reducing the risks of HIV infection from sex and injecting drug use. Neither prison service initially acknowledged that sex or drug-taking took place within its establishments, but by the late 1980s, advisory bodies were making recommendations that challenged this stance. Guidelines from the WHO and the Council of Europe stated that condoms should be provided to prisoners, that facilities for safer injecting should be made available, whether in the form of needle exchanges or disinfectant, and that methadone should be prescribed on a long-term basis as an alternative to heroin. With the exception of needle exchanges in prisons, these proposed policies were endorsed by various groups in England & Wales including the Advisory Council on the Misuse of Drugs, the Prison Reform Trust, the prison service AIDS Advisory Committee, set up in 1986 and comprising both prison staff and external HIV/AIDS experts, and the AIDS and Prisons Forum organised by the National AIDS Trust.^51^ In the Republic of Ireland, however, expert bodies and voluntary pressure groups were fewer in number and less influential. Groups addressing gay health and addiction were growing, but did not yet have the access and power to shape policy. Drug agencies were providing needle exchanges and long-term methadone in the wider community, but on a relatively unofficial footing which hindered the ability of the drug treatment community to speak out. An Advisory Committee on Communicable Diseases in prisons was set up in 1990 in response to the turmoil prompted by HIV/AIDS, but only two of its 13-strong membership were from outside the Department of Justice and it declined to recommend condoms or needle exchanges, focusing instead on the need to regularise medical practices within prisons and the ongoing issue of segregation.^52^ Irish prisons remained isolated, and discussion of HIV/AIDS policy at the national level was still limited. External pressures on prisons were not yet being felt.

Recommendations that condoms be made available to male prisoners prompted some debate in medical journals and campaigning amongst HIV/AIDS advocacy bodies in England & Wales, but the prison service steadfastly opposed the idea.^53^ Civil servants and management maintained that prisons were not private places for the purposes of the 1967 Sexual Offences Act and thus sexual activity between men was illegal; furthermore, many prisons housed young men below the age of consent for homosexuality. The provision of condoms therefore risked endorsing or encouraging criminal activities. Additional objections were added to this: that condoms could be used for smuggling or in making weapons, and that they were not of proven efficacy in the case of anal sex. Home Affairs Minister David Mellor advised that the Home Office ‘cannot be certain that a change of policy would not encourage significantly more sexual activity of a kind which, even with a condom, carries a particularly high risk of HIV infection’.^54^ Many argued that the problem was being blown out of proportion in any case. One senior civil servant felt strongly that the debate in the late 1980s was very much ‘based on presumptions about the degree of homosexuality that might occur in prisons’ rather than facts. Illustrating that even prisoners felt that it was getting out of hand, he recounted hearing that ‘a prisoner had expressed in a humorous way his concern about all of this stuff in the press about homosexuality in prisons, and said something like “Next thing they’ll be making it compulsory”’.^55^ If prisoners found these swirling concerns about sex and HIV/AIDS in prisons excessive, then surely external commentators should take heed.

Condoms for prisoners remained contentious throughout the 1990s. External recommendations and criticisms persisted, and one health worker recalled that there were ‘tactical games played at senior level, where people were trying to move the boundaries as much as possible’.^56^ The HIV/AIDS guidance for prisons issued in 1991 noted that Ministers ‘have not been convinced that making condoms available for use in prison would be appropriate or helpful’, but observed and implicitly endorsed the fact that some establishments were making condoms available to prisoners on release. The AIDS and Prisons Forum, chaired by a retired Deputy Director General of the prison service and attended by serving staff, obtained legal advice on the status of sex in prisons and the responsibilities of the prison service, which offered support to the view that condoms should be made available. Under cover of a 1994 ‘Dear Doctor Letter’ (DDL), correspondence sent directly from the Director of Prison Medical Services to all prison doctors, Dr Wool informed colleagues of the ‘first recorded case of HIV transmission within the English Prison Service’, which was ‘by sexual contact’. ‘Doctors have the clinical freedom to prescribe pharmaceutically or otherwise to protect the health of individual prisoners’, the DDL concluded. ‘Legal advice is that doctors who so prescribe for their patients with appropriate advice, in the exercise of their clinical judgment, could expect protection in law for doing so’.^57^ The following year, Home Secretary Michael Howard blocked the wishes of the HMPS’s AIDS Advisory Committee and the Prisons Board to make condoms freely available within prisons, as part of a broader effort to appear ‘tough on crime’ which included vetoing the provision of televisions in prison cells.^58^ In response, Dr Wool’s oblique authorisation to issue condoms was made explicit in a further DDL.^59^ Prompted at least in part by evidence of HIV transmission within prisons and fears of legal action as well as insistent pressure from external agencies such as the AIDS and Prisons Forum, this policy received the endorsement of the courts in 1999 and remains in place today.^60^

Ongoing calls to permit prisoners access to condoms reflected the readiness with which condoms had been accepted as a valuable public health measure across most of British society. They also reflected the continuing scrutiny of a variety of external bodies which kept up pressure on the prison service to reconsider its approach, and the growing numbers of health promotion workers, HIV/AIDS co-ordinators, and genito-urinary consultants who were beginning to work in prisons.^61^ This influx had been prompted in part by the funding available to local councils, health authorities and charities for specialists in HIV/AIDS. By the 1990s, it was also flowing from the encouragement given to the prison service to purchase services rather than trying to provide them directly. This was part of a broader move to lessen the direct involvement of government departments in the management of state services, represented by the introduction of Executive Agencies to run services at arm’s length from their government departments. HMPS became an Executive Agency in 1993. As the prison service inched towards greater collaboration with the NHS and with voluntary bodies, some of the hard distinctions between prisons and the wider community were difficult to maintain.

Official opposition to condoms was maintained in the mid-1990s by the particularly staunch commitment of ministers and government to focus upon security and punishment in prisons, at the expense of efforts to achieve equivalence with community services. The gap between the prison medical service and the NHS mattered less here than the politicisation of criminal justice. However, criticisms from external bodies and internal concerns about the spread of infection and resultant legal action prompted penal policy makers to once again follow the path of a permissive policy that relied upon the decision making of its doctors. As with the VIR, the presence of senior medical staff within the prison service with some independence and authority made this possible, but also allowed for considerable variation in implementation. In some instances, doctors interpreted the guidance extremely narrowly; in others, they remained unaware of its existence.

In the Republic of Ireland, there was virtually no discussion about changing policy to allow the provision of condoms to prisoners. It was mentioned within the Department of Justice in the aftermath of the first HIV diagnoses in Mountjoy and there was reportedly some caveated support, but it proved too controversial for the state to pay for and provide condoms when homosexuality remained entirely illegal and the sale of condoms to those at liberty was still restricted.^62^ By 1993, when the Advisory Committee on Communicable Diseases in Prison published its report, it simply stated that ‘bearing in mind the legal position and the lack of evidence that anal intercourse between prisoners is common in prisons’, they ‘considered it inappropriate’ for condoms to be provided.^63^ In the 1990s, laws and mores surrounding homosexuality and contraceptives were beginning to change, with homosexuality decriminalised and legislation surrounding contraception relaxed, but these changes had been fiercely contested.^64^ There was little appetite even amongst those few interested in penal reform for raising the highly charged issue of sex amongst prisoners. It was not mentioned by the Mountjoy Visiting Committees in their increasingly critical annual reports, and does not feature prominently in the memories of those involved in prison work. Some staff did take condoms in to Mountjoy, but this was on a small scale and official policy remained silent on the subject.^65^ As the first Head of Nursing within the prison service recalled, it was only in the 2000s that she and colleagues managed to establish the availability of condoms on request.^66^

One Dublin-based addiction worker remarked that ‘everybody found it much easier to talk about drug use and injecting than safer sex’.^67^ This reflected how HIV/AIDS was perceived, as well as cultural and political anxieties around sex, sexuality and contraception. In the Republic of Ireland, more than half of all positive test results for HIV in the 1980s and early 1990s were amongst injecting drug users. This compared to just 10–12 per cent in the UK, where homosexuality was the most commonly stated route of transmission.^68^ It was therefore clean needles or methadone for drug users in prison rather than condoms for their sexually active cellmates that the Irish prison service tackled first. Dublin’s ‘opiate epidemic’ of the early 1980s appeared to have been intensely localised: particular estates and even families were extremely severely affected by both heroin and HIV/AIDS. As one former drug user and addiction worker explained, ‘I’ve three cousins that died of AIDS, just in the one family. … I think they died in the same year. And a lot of their friends, all around the same age, they died’.^69^ This heavy toll in the late 1980s and early 1990s encouraged the city’s health authorities to take action. As has been argued elsewhere, drug services in Dublin moved away from an emphasis upon complete abstinence and began to pursue methods of harm minimisation including providing sterile needles, disseminating information about safer injecting practices and prescribing oral methadone on a long-term basis as an alternative to heroin, albeit somewhat covertly at first.^70^ New clinics were set up around the city, and the problem of illicit drug use rose rapidly up the policy agenda.

As with the emergence of HIV/AIDS and the subsequent policy of segregation, the problem of drug use appeared to be firmly concentrated in the Mountjoy prison complex in Dublin, and new policies and practices were adopted there first. Prior to the mid-1990s, there had been very little by way of services or treatment for addiction in Mountjoy, other than advice from probation and welfare officers, chaplains and one or two addiction treatment services that were granted access to talk to inmates and encourage them to seek out their services.^71^ The two visiting GPs at Mountjoy were not particularly interested in the field of addiction, and methadone was not prescribed.^72^ New policies emerged only after strategies had been tested out on the ground. Innovations were not always taken up: attempts by some prison officers to introduce bleach into Mountjoy for inmates to clean injecting equipment did not catch on.^73^ Changes driven by doctors stood a greater chance of success, reflecting the potential for medical voices to carry significant weight within prison systems.

This also pointed towards the growing importance and influence of medical expertise within the Irish prison system in particular, as armed conflict on the island lessened and attention to more everyday matters of crime and punishment grew. Methadone was quietly introduced and prescribed to prisoners in the separation unit, ostensibly only during detoxification but in practice on a longer-term basis as well, and this continued in the new Medical Unit when it opened in 1993. In 1996, a new drug treatment programme was launched and a new GP with expertise in addiction and experience in prescribing long-term methadone was hired alongside agency nurses, also with knowledge and experience of community practice.^74^ Treatment was on a very small scale at first with only nine beds available at a time, but the wider prescribing of methadone across the men’s prison was gradually adopted as well. Following the recruitment of a pharmacist, increasing numbers of external organisations establishing links with prisons, and the first research into blood-borne viruses in Irish prisons, the Department of Justice set up a multi-disciplinary steering group in 1999 to consider the way forward for its addiction policies. The emphasis was on continuity of care, replicating community services in prisons as much as possible, and medical views were coming to dominate.^75^

Policy and practice surrounding drug addiction in Irish prisons changed markedly.^76^ This was partly because HIV/AIDS remained a significant concern, not least because of the longevity and legacy of the separation unit. However, in the absence of many external pressure groups or advisory bodies in the Republic of Ireland, change relied heavily upon influences from within the prison system and within Mountjoy, where innovations had already been adopted with little public discussion and policy changes were required simply to keep pace. This was not unusual insofar as drug policy was concerned, but represented something of a change for penal policy, and a slight loosening of the grip of the Department of Justice over day to day activities. It coincided with a period of restructuring and reform for prisons as a whole in the 1990s, facilitated by greater public and political interest in matters of law and order, growing awareness and discussion of human rights, and progress towards the ending of the Troubles, which began to alleviate some security concerns and encouraged movement towards greater openness. The Department of Justice produced its first strategy document for prisons in 1995, and in 1999 a distinct Irish Prison Service was created with a named Director and publicly stated goals and priorities. Greater openness was also encouraged by some senior staff, who realised the value of external research, recommendations, and scrutiny when it came to driving change. Adjustments to drug treatment policy also reflected the growing involvement of community agencies, who brought with them the practices and approaches that had been adopted elsewhere, and greater numbers of medical professionals including qualified nurses, who shaped policy surrounding matters of health.

Policy relating to harm minimisation in England & Wales changed less. Indeed, by the mid-1990s and the compromise over condoms, the issue of HIV/AIDS had faded. Rates of HIV/AIDS amongst prisoners and injecting drug users were comparatively low.^77^ Treatment for opioid addiction was erratic: a survey of prison heads of healthcare undertaken by the Director of Medical Services in 1991 found that of the respondents, 20 per cent of establishments offered methadone for detoxification purposes and 7 per cent for maintenance, but elsewhere prison doctors remained entirely opposed to methadone and offered no treatment beyond the management of withdrawal symptoms.^78^ Again, policies were permissive and relied upon medical discretion, with overall responsibility for treatment in the hands of the Director of Nursing by the mid-1990s. In the case of harm minimisation, the divide between disciplinary and medical approaches to addiction weakened the influence of its advocates, and operational managers and doctors alike could ignore it in favour of meeting other objectives. Specific targets became an increasingly common feature of prison management over the 1990s, with the arrival of Key Performance Indicators and a focus upon offender management, particularly risk management.^79^ One such target stemmed from mandatory drug testing, introduced in 1995. Mandatory drug testing in prisons epitomised the political will during these years to appear tough on crime and punitive, and to tackle the problem of reoffending which appeared to be closely associated with illicit drug use. As the Chief Inspector of Prisons at this time recalled, prisons could test inmates they knew to be drug-free which would meet their targets, but would not address addiction at all. Visiting a cell one day he found ‘a person with 9 pieces of paper stuck on the wall. I said, “What are those?” He said, “That’s my drug free certificates: they know I don’t use, and if you come back in 2 weeks, I’ll have a 10^th^, because they just test me to make their figures look good”’.^80^ In this context, reducing harms associated with drugs had to compete with pressures to reduce the visibility of drugs and to meet new management targets, and policies flowing through medical channels were particularly limited.

The introduction of disinfecting tablets for cleaning injecting equipment also faced difficulties in England & Wales: despite repeated recommendations and a successful pilot in the late 1990s, it was not rolled out more widely for some time and continued to be beset with problems.^81^ Nevertheless, some movement towards the greater integration of prisons with community services and policies continued, with gradual impact upon policy. Prison policy on drugs was included in national strategies for the first time, with public health bodies encouraged to incorporate prisons in their plans, and research into the extent of psychiatric morbidity in prisons and the efficacy of methadone prescribing added further weight to calls for the prison service to open its doors to NHS services and external expertise.^82^ NHS staff were seconded to the prison service to develop policy and practice, and new standards for services for substance misusers were introduced.^83^ The political temperature remained important, especially as concerns around blood-borne viruses continued to subside, but the greater permeability of prisons at the end of the 1990s meant that penal policy was becoming more closely aligned to national policy.

## Conclusions

Histories of HIV/AIDS are beginning to analyse the events of the 1980s and 1990s from new perspectives. The reactions of prisons provide a useful lens through which to reexamine policy making and its interpretation during those decades. Broadly characterised, prison policy in England does partly fit the model of a ‘liberal consensus’, but this is less true for prison policy in Ireland. However, in both cases the question of prison policy in relation to HIV/AIDS reflects the social conservatism with which the 1980s is often associated. Prison policy making also illustrates the importance of institutional structures, histories and knowledge as well as the broader canvas of social, cultural and political contexts onto which HIV/AIDS emerged. This is also demonstrated through the comparison here of two distinct jurisdictions, close in terms of geography and population flow but at a distance from one another in other respects. Differences in national responses to HIV/AIDS were important to prison policy making, but so too were localised concerns and preoccupations, the nature and extent of external scrutiny, and the presence and influence of a variety of experts within decision making circles, particularly medical professionals. This adds to our understanding of the history of prisons over this period, which has at times been over-simplified as an era of endless crisis caused by overcrowding and understaffing. The case of the Republic of Ireland also adds nuance to existing periodisations and characterisations of HIV/AIDS policy making, suggesting more muted and even covert activities over the 1980s and into the 1990s. There is undoubtedly scope for much more work on Irish reactions to, and experiences of, HIV/AIDS.

Pre history was important here. When compared to national policies, penal policies in response to HIV/AIDS could appear excessive, as in the case of segregation, or insufficient, as with the availability of condoms. The long-standing isolation of the Irish Department of Justice, its preoccupation with security, and its relative inexperience with issues such as overcrowding, as well as the extent and geographical focus of its first encounter with HIV/AIDS, prompted the creation of the segregation unit for prisoners with HIV/AIDS. The absence of external expertise or scrutiny helped it to endure, with interested voluntary groups and individual experts few in number. The absence of medical professionals within the Irish penal system was also significant, as was the presence of senior medical staff on the other side of the Irish sea. Policies within HMPS were influenced by medical and other expert views, both internally and coming from external advisory bodies and committees. But, and to the detriment of implementation, they also relied heavily upon medical discretion. This indicates the impact of greater levels of local independence and less central control within HMPS than was the case for the Irish Prison Service.

Policies in the Republic began to change as the prison service itself began to expand and open up over the 1990s. This was a period of change for the nation more broadly, with shifts in attitudes towards homosexuality and contraception that would go on to have an impact upon prisons. Also key was a lessening of sectarian conflict and greater criticism of national institutions such as the Church over this period: the Department of Justice was not immune from criticism, and was encouraged by this changing context to adjust its management and priorities for the prison system. HIV/AIDS became one such priority, and the involvement of new drug agencies and medical professionals in prisons helped to shift practices on the ground. Advice and recommendations from external bodies finally began to emerge, providing not only support for those within the service who wanted to make the case for change, but also the promise of further public scrutiny. The creation of the Irish Penal Reform Trust in 1994 was indicative of this new scrutiny. Changes in England & Wales were less marked, interrupted by the imposition of ‘tough on crime’ rhetoric from government and de-prioritised as targets focused on offender management rose and HIV/AIDS dropped down the agenda, but external scrutiny did not disappear. Furthermore, as the prison service negotiated partial privatisation and its new status as a purchaser of services, ever-closer union with the NHS and other external providers drew nearer. NHS staff as well as HIV/AIDS co-ordinators and drug agency workers were to be found inside prisons in greater numbers, bringing with them the policies and standards of the wider community. HIV/AIDS therefore coincided with a lessening of the isolation of prison services, acting as one amongst many reminders that prison walls were not impermeable. In the Republic of Ireland, this was particularly pronounced, as the secrecy surrounding the prison system as a whole began to subside, but it was apparent in England & Wales as well. As stronger links were forged between prisons and social care providers, including health services and voluntary bodies, penal policy moved closer towards parity with national policy as the new century approached.

## Footnotes

1 Although HIV and AIDS are now recognised as distinct medical conditions, they were often confused and conflated during the period under examination, and policy responses tended to treat them as one and the same issue. We therefore use the term ‘HIV/AIDS’.

2 Gary P. Wormser, ‘Acquired Immunodeficiency Syndrome in Male Prisoners: New Insights into an Emerging Syndrome’, *Annals of Internal Medicine*, 1983, 98, 297; Theodore M. Hammett and Monique Sullivan, *AIDS in Correctional Facilities: Issues and Options* (Washington, DC: US Department of Justice, 1986); Advisory Council on the Misuse of Drugs, *AIDS and Drug Misuse: Part One* (London: HMSO, 1988) ch. 8; *HIV, AIDS and Prisons* (London: Prison Reform Trust, 1988); T. Harding, ‘HIV Infection and AIDS in the Prison Environment: A Test Case for the Respect of Human Rights’, in John Strang and Gerry V. Stimson, eds, *AIDS and Drug Misuse: The Challenge for Policy and Practice in the 1990s* (Abingdon: Routledge, 1990), 197–210.

3 For example, Council of Europe, Parliamentary Assembly, ‘Recommendation 1080 on a Coordinated European Policy to Prevent the Spread of AIDS in Prisons’, 1988. <https://www1.umn.edu/humanrts/instree/recommendation1080.html>, accessed 8 April 2016.

4 For example, Mildred Blaxter, *AIDS: Worldwide Policies and Problems* (London: Office of Health Economics, 1991); B. A. Miztal and D. Moss, eds, *Action on AIDS: National Policies in Comparative Perspective* (New York: Greenwood Press, 1990).

5 For example, Simon Watney, *Practices of Freedom: Selected Writings on HIV/AIDS* (Durham, NC: Duke University Press, 1994).

6 Daniel M. Fox, Patricia Day and Rudolf Klein, ‘The Power of Professionalism: Policies for AIDS in Britain, Sweden, and the United States’, *Daedalus*, 1989, 118, 93–112.

7 Virginia Berridge, *AIDS in the UK: The Making of Policy, 1981–1994* (Oxford: Oxford University Press, 1996).

8 Virginia Berridge and Philip Strong, eds, *AIDS and Contemporary History* (Cambridge: Cambridge University Press, 1993).

9 The Chief Medical Officer of England used such comparisons in support of his own preferred tactics, which favoured confidentiality and non-compulsion: Donald Acheson, *One Doctor’s Odyssey: The Social Lesion* (Bury St Edmunds: Arima Publishing, 2007), 62. On the uses of history, see also Roy Porter, ‘History Says No to the Policeman’s Response to AIDS’, *British Medical Journal*, 1986, 293, 1589–90; Elizabeth Fee and Daniel M. Fox, eds, *AIDS: The Burdens of History* (Berkeley, CA: Berkeley University Press, 1988).

10 For example, *Report to the Irish Government on the Visit to Ireland Carried out by the European Committee for the Prevention of Torture and Inhuman or Degrading Treatment or Punishment*, (Strasbourg: Council of Europe, 1995); Oonagh O’Brien and Stevens, *A Question of Equivalence: A Report on the Implementation of International Guidelines on HIV/AIDS in Prisons of the European Union* (London: Cranstoun Drug Services, 1997); T.W. Harding, ‘AIDS in Prison’, *The Lancet*, 1987, 330, 1260–63; Paul J. Turnbull, Kate A. Dolan and Gerry V. Stimson, *Prisons, HIV, and AIDS: Risks and Experiences in Custodial Care* (Horsham: AVERT, 1991).

11 England & Wales have a single, integrated penal system, distinct from that of Scotland and Northern Ireland.

12 Examples of historical studies of HIV/AIDS include Matt Cook, ‘“Archives of Feeling”: The AIDS Crisis in Britain 1987’, *History Workshop Journal*, 2017, 83, 51–78; Richard A. McKay, ‘“Patient Zero”: The Absence of a Patient’s View of the Early North American AIDS Epidemic’, *Bulletin of the History of Medicine*, 2014, 88, 161–94; Anthony Michael Petro, *After the Wrath of God: AIDS, Sexuality, and American Religion* (Oxford: Oxford University Press, 2015); Cheryl Ware, ‘“Time to Speak Out”: The 1980s Australian Gay Press and Personal Accounts of Living with HIV’, *Journal of Australian Studies*, 2017, 41, 1–15.

13 For example, Jonathan Bell, ‘Rethinking the “Straight State”: Welfare Politics, Health Care, and Public Policy in the Shadow of AIDS’, *Journal of American History*, 2018, 104, 931–52; ‘HIV/AIDS and U.S. History’, *Journal of American History*, 2017, 104, 431–60, at 444, and the EUROPACH project, subtitled ‘Disentangling European HIV/AIDS Policies: Activism, Citizenship and Health’: <https://www.euroethno.hu-berlin.de/de/forschung/projekte/europach>.

14 ‘HIV/AIDS and U.S. History’, 444. For the growing interest in prisons as sites of health care and health policy, see the Wellcome Trust-funded project ‘Prisoners, Medical Care and Entitlement to Health in England and Ireland, 1850–2000’, running from 2015 to 2020, and Dan Healey, ‘Lives in the Balance: Weak and Disabled Prisoners and the Biopolitics of the Gulag’, *Kritika: Explorations in Russian and Eurasian History*, 2015, 16, 527–56.

15 Recordings, transcripts or notes from both sets of interviews are available from the Archives of the London School of Hygiene & Tropical Medicine.

16 Virginia Berridge, ‘Researching Contemporary History: AIDS’, *History Workshop*, 1994, 38, 228–34.

17 See, for example, Janet Weston and Virginia Berridge, eds, *HIV/AIDS and the Prison Service of England & Wales, 1980s–1990s: Transcript of a Witness Seminar* (London: London School of Hygiene and Tropical Medicine, 2017). Dr Hilary Pickles, head of the AIDS Unit in the 1980s, participated and contributed freely but had been prevented from giving an interview in the 1980s.

18 Brian Girvin, ‘Church, State, and Society in Ireland since 1960’, *Éire-Ireland*, 2008, 43, 74–98.

19 Michael Cronin, ‘Small Worlds and Weak Ties: Ireland in the New Century’, *Journal of Irish Studies*, 2007, 22, 63–73, at 65.

20 See Diarmaid Ferriter, *Occasions of Sin: Sex and Society in Modern Ireland* (London: Profile Books, 2009) for one such equivocation of Ireland’s former exceptionalism and the speed of change.

21 Mike Tomlinson, ‘Imprisoned Ireland’, in Vincenzo Ruggiero, Mick Ryan and Joe Sim, eds, *Western European Penal Systems: A Critical Anatomy* (London: Sage Publications, 1995), 194–227, at 195; Eoin O’Sullivan and Ian O’Donnell, *Coercive Confinement in Ireland: Patients, Prisoners and Penitents* (Manchester: Manchester University Press, 2012); Ian O’Donnell, ‘Stagnation and Change in Irish Penal Policy’, *The Howard Journal of Criminal Justice*, 2008, 47, 121–33; Mary Rogan, *Prison Policy in Ireland: Politics, Penal-Welfarism and Political Imprisonment* (Abingdon: Routledge, 2011).

22 David Garland, *Punishment and Welfare: A History of Penal Strategies* (Aldershot: Gower, 1987); Joe Sim, *Medical Power in Prisons: The Prison Medical Service in England 1774–1989* (Milton Keynes: Open University Press, 1990); Janet Weston, ‘Sexual Crimes, Medical Cures: The Development of a Therapeutic Approach toward Sexual Offenders in English Prisons, c.1900–1950’, *Canadian Journal of History*, 2014, 49, 395–422.

23 A useful summary of staffing can be found in the Social Services Committee minutes, ‘Prison Medical Service: Minutes of Evidence, 20 November 1985: Dr John Kilgour and Mr Peter Barker’, 1–2, 5, and in John L. Kilgour, ‘The Prison Medical Service in England and Wales: A Commentary from the Director of the Prison Medical Service’, *British Medical Journal*, 1984, 288, 1603–5.

24 For example, the 1979 Committee of Inquiry into the United Kingdom Prison Services, the 1984 Home Office Social Services Committee investigation into the Prison Medical Service; organisations including Radical Alternatives to Prison, INQUEST, Women in Prison, and the Prison Reform Trust; Les Blair [dir.], ‘The Prisoners’ Tale’, *Law and Order* (BBC Two, 1978); Mike Fitzgerald and Joe Sim, ‘Legitimating the Prison Crisis: A Critical Review of the May Report’, *The Howard Journal of Criminal Justice*, 1980, 19, 73–84; and Dr Richard Smith’s 1983 series of articles on the prison medical service in the *British Medical Journal*.

25 Richard Smith, ‘Disorder, Disillusion, and Disrepute’, *British Medical Journal*, 1983, 287, 1521–3; *Prison Medicine: Ideas on Health Care in Penal Establishments* (London: Prison Reform Trust, 1985); Sim, *Medical Power in Prisons*.

26 Rogan, *Prison Policy in Ireland*; Paul O’Mahony, *The Irish War on Drugs: The Seductive Folly of Prohibition* (Manchester: Manchester University Press, 2008), 87–9; John Maher, ‘Mountjoy Facing Disaster, Says Visiting Committee Report’, *Irish Times*, 8 May 1996, 1.

27 Roy D. King and Kathleen McDermott, *The State of Our Prisons* (Oxford: Oxford University Press, 1995); Mick Ryan, *Penal Policy and Political Culture in England and Wales: Four Essays on Policy and Process* (Winchester: Waterside Press, 2003); Trevor Jones and Tim Newburn, *Policy Transfer and Criminal Justice: Exploring US Influence over British Crime Control Policy* (Maidenhead: Open University Press, 2007); Michael Cavadino and James Dignan, *Penal Systems: A Comparative Approach* (London: Sage Publications, 2006), ch. 4.

28 Fiona Smyth, ‘Cultural Constraints on the Delivery of HIV/AIDS Prevention in Ireland’, *Social Science and Medicine*, 1998, 46, 661–72; Ann Marie Nolan, ‘Marriage is Not an Anti-viral Agent’: The Transformation of Sexual Health Policy in the Initial Decade of HIV/AIDS in Ireland’ (PhD thesis, Trinity College Dublin, 2014); David Kilgannon, ‘How to Survive a Plague: AIDS Activism and Ireland, 1983–89’ (MA thesis, University College Dublin, 2015). For an overview of the historiography of Irish medicine and health, see Catherine Cox, ‘A Better Known Territory? Medical History and Ireland’, *Proceedings of the Royal Irish Academy*, 2013, 113C, 341–62.

29 ‘Ban on Movement at Aids scare Gaol’, *Guardian*, 6 February 1985, 3.

30 Telephone conversation with Rosemary Wool; interview with former part-time Prison Medical Officer Dr Pat Lush, 1 November 2016; interview with former prison Hospital Chief Officer, John Ramwell, 12 January 2017.

31 Social Services Committee, ‘Prison Medical Service: Minutes of Evidence, 5 February 1986: Dr John Kilgour and Mr Peter Barker’, 110.

32 For an example of a medical officer’s intervention that was remembered as unsuccessful, see interview with former HMPS Prison Governor, Stephen Twinn, 18 January 2017.

33 Berridge, *AIDS in the UK*, 55–75.

34 HM Prison Service, ‘HM Prison Service Policy—Circular Instruction 30/1991’, Annex D.

35 Prison Reform Trust, *HIV, AIDS and Prisons*; *HIV, AIDS and Prisons: Update* (London: Prison Reform Trust, January 1991); ‘HIV in Prisons’, *The Lancet*, 1991, 337, 546; *Prison Disturbances April 1990: Report of an Inquiry by the Rt. Hon Lord Justice Woolf and His Honour Judge Stephen Tumin* (London: HMSO, 1991), 333–5; Advisory Council on the Misuse of Drugs, *AIDS and Drug Misuse: Part Two* (London: HMSO, 1989); ‘Comment’, *Prison Service Journal*, 1990, 78, 1.

36 HM Prison Service Policy—Circular Instruction 30/1991.

37 Interview with anonymous public health professional, 18 July 2017. The use of segregation for operational reasons was also mentioned critically in T. W. Harding, ‘AIDS in Prison’, 1261.

38 Una Padel, *HIV Education in Prisons* (London: Health Education Authority/SCODA, 1992), 5. On Wandsworth, see also interview with anonymous public health professional, 18 July 2017.

39 *Report of the Director of Health Care for Prisoners* (London: HM Prison Service, April 1992), 34; Stephen Tumin, *Report of Her Majesty’s Chief Inspector of Prisons 1993–1994* (London: HMSO, 1994), 43.

40 Berridge, *AIDS in the UK*; Karen Duke, *Drugs, Prisons and Policy-Making* (London: Palgrave Macmillan, 2003).

41 Interview with former Deputy Director General of HMPS Tony Pearson, 21 February 2017.

42 Interview with former probation and welfare officer Julian Pugh, 27 October 2016; Paul O’Mahony and Thomas Gilmore, *Drug Abusers in the Dublin Committal Prisons: A Survey* (Dublin: Department of Justice, 1982).

43 Interview with founders of the Ana Liffey Drug Project Father Frank Brady and Mara de Lacy, 17 November 2016; interview with former Mountjoy Governor John Lonergan, 16 February 2017; ‘Death in Mountjoy’, *Today Tonight*, RTE One, 21 January 1986. See also Paul Howard, *The Joy, Mountjoy Jail: The Shocking, True Story of Life Inside* (Dublin: O’Brien Press, 1996), 34.

44 Interview with Former Mountjoy Governor John Lonergan; *Annual Report on Prisons and Places of Detention: 1986* (Dublin: Department of Justice, 1987). On the longevity of segregation, see O’Brien and Stevens, *A Question of Equivalence*; Council of Europe, *Report to the Irish Government on the Visit to Ireland,* 1995; Interview with former Director of Prison Medical Services Dr Enda Dooley, 16 February 2017.

45 Interview with Former Mountjoy Governor John Lonergan; interview with probation and welfare officers, 15 February 2017; interview with former Director of Prison Medical Services Dr Enda Dooley.

46 Maev-Ann Wren, *Unhealthy State: Anatomy of a Sick Society* (Dublin: New Island, 2003).

47 Interview with probation and welfare officers; Report of the Mountjoy Visiting Committee, in *Annual Report on Prisons and Places of Detention: 1993* (Dublin: Department of Justice, 1996), 60–1; Report of the Mountjoy Visiting Committee, in *Annual Report on Prisons and Places of Detention: 1994* (Dublin: Department of Justice, 1997), 62; *Report of the Advisory Committee on Communicable Diseases in Prison*, (Dublin: Department of Justice, 1993), 14–16.

48 *Report of the Advisory Committee on Communicable Diseases in Prison*, 9, Appendix II.

49 The policy also led John Lonergan to maintain that segregation had been abandoned in the late 1980s for all but a handful. Interview with former Mountjoy Governor John Lonergan.

50 *Annual Report on HIV/AIDS for 1993* (Dublin: Department of Health, 1993), 12.

51 *HIV and AIDS: A Multidisciplinary Approach in the Prison Environment* (London: Directorate of Health Care Services, 1990); *Review of HIV and AIDS in Prison* (London: AIDS Advisory Committee, 1995); Di Robertson, *HIV and Custody: Risk Reduction and Health Promotion in Custodial Settings* (London: Health Education Authority/National AIDS Trust, 1995), 26; Weston and Berridge, *HIV/AIDS and the Prison Service of England & Wales*.

52 *Report of the Advisory Committee on Communicable Diseases in Prison*. See also *National AIDS Strategy Committee: Reports and Recommendations of the Subcommittee on Care and Management of Persons with HIV/AIDS* (Dublin: Department of Health, 1992), which focused on segregation in prisons.

53 G. Neil Conacher, ‘AIDS, Condoms, and Prisons’, *The Lancet*, 1988, 332, 41–2; A McMillan, ‘HIV in Prisons: Action, Research and Condoms Needed’, *British Medical Journal*, 1988, 297, 873–4; Interview with Steve Morton, formerly of the Lambeth Health Promotion Unit, 28 November 2016; Simon Garfield, *The End of Innocence: Britain in the Time of AIDS* (London: Faber & Faber, 1995), 181–2.

54 Weston and Berridge, *HIV/AIDS in the 1980s and 1990s*; interview with anonymous former prison governor, 7 December 2016; interview with Steve Morton; Len Curran, *Arguments for and against the Issue of Condoms in Prison as a Preventive Measure in the Fight against HIV & AIDS* (London: HM Prison Service, 1991); Parliamentary Debates (Commons): Written answers, 167, 12 February 1990, c7W.

55 Interview with former Director of Operational Policy for the Prison Service Sydney Norris, 8 February 2017. See also interview with former prison Hospital Chief Officer, John Ramwell.

56 Interview with Steve Morton.

57 The 1994 DDL is quoted in full in Parliamentary Debates (Commons): Written answers, 244, 26 May 1994, 271.

58 See Yvonne Jewkes, ed., *Handbook on Prisons* (Abingdon: Routledge, 2013), 457.

59 AIDS Advisory Committee; Weston and Berridge, *HIV/AIDS in the 1980s and 1990s*. The 1995 DDL is quoted in full in *R v Secretary of State for Home Department ex parte Glen Fielding* [1999] EWHC (Admin) 641.

60 *R v Secretary of State for Home Department ex parte Glen Fielding*. Criticisms of the policy include Advisory Council on the Misuse of Drugs, *Drug Misusers and the Criminal Justice System, Part Three: Drug Misusers and the Prison System: An Integrated Approach* (London: HMSO, 1996), 104; British Medical Association Foundation for AIDS, *Prescribing of Condoms in Prison: Survey Report* (London: British Medical Association, 1997); All Party Parliamentary Group on AIDS, *The UK, HIV and Human Rights: Recommendations for the next Five Years* (London: HMSO, 2001), 28; Ailsa Stevens, *Sex in Prison* (London: Howard League for Penal Reform, 2015).

61 Interview with Roger Howard, formerly of the National Association for the Resettlement of Offenders (NACRO) and the Standing Conference on Drug Abuse (SCODA), 27 June 2017; Interview with Steve Morton.

62 Interview with former Mountjoy Governor John Lonergan.

63 *Report of the Advisory Committee on Communicable Diseases in Prison*, 30.

64 Wren, *Unhealthy State*, 125.

65 Interview with former probation and welfare officer Julian Pugh; interview with anonymous drug addiction worker in Dublin, 15 February 2017.

66 Interview with former Head of Nursing in the Irish Prison Service, Frances Nangle Connor, 15 November 2016.

67 Interview with anonymous drug addiction worker. More generally, see Diarmaid Ferriter, *Occasions of Sin*, 425; Diarmaid Ferriter, *The Transformation of Ireland, 1900–2000* (London: Profile Books, 2004); Nolan, ‘Marriage is Not an Anti-viral Agent’; Smyth, ‘Cultural Constraints on the Delivery of HIV/AIDS Prevention in Ireland’.

68 *National AIDS Strategy Committee: Reports and Recommendations of the Subcommittee on Care and Management of Persons with HIV/AIDS*, 9; Garfield, *The End of Innocence*, 361.

69 Interview with drug addiction worker Paul Hatton, 16 November 2016. On heroin in 1980s Dublin, see Geoffrey Dean, Michael Kelly, Aileen O’Hare, Grainne Kelly and Aideen O’Connor, ‘The Opiate Epidemic in Dublin 1979-1983’, *Irish Medical Journal*, 1985, 78, 107–10; Geoffrey Dean, Aileen O’Hare, Aideen O’Connor, Michael Kelly and Grainne Kelly, ‘The “Opiate Epidemic” in Dublin: Are We over the Worst?’, *Irish Medical Journal*, 1987, 80, 139–42; interview with founders of the Ana Liffey Drug Project Father Frank Brady and Mara de Lacy; Fergus O’Kelly, ‘Heroin Abuse in an Inner-city Practice’, *Irish Medical Journal*, 1986, 79, 85–7.

70 Shane Butler, ‘The Making of the Methadone Protocol: The Irish System?’, *Drugs: Education, Prevention and Policy*, 2002, 9, 311–24; Shane Butler, *Alcohol, Drugs, and Health Promotion in Modern Ireland* (Dublin: Institute of Public Administration, 2002); Shane Butler and Paula Mayock, ‘“An Irish Solution to an Irish Problem”: Harm Reduction and Ambiguity in the Drug Policy of the Republic of Ireland’, *International Journal of Drug Policy*, 2005, 16, 415–22.

71 Interview with probation and welfare officers.

72 Indeed, consensus has it that they were not particularly interested in treating prisoners at all. Interview with former Mountjoy Governor John Lonergan; interview with probation and welfare officers David Williamson *et al*.; interview with former Director of Prison Medical Services Dr Enda Dooley; interview with Mountjoy addiction specialist, Dr Des Crowley, 16 November 2016.

73 Interview with anonymous drug addiction worker; interview with former probation and welfare officer Julian Pugh.

74 Interview with anonymous drug addiction worker.

75 Interview with former probation and welfare officer Tony Carlin, 16 November 2017; interview with Mountjoy addiction specialist Dr Des Crowley; Des Crowley, ‘The Drug Detox Unit at Mountjoy Prison: A Review’, *Journal of Health Gain*, 1999, 3, 17–19; S. Allwright and others, *Hepatitis B, Hepatitis C and HIV in Irish Prisoners: Prevalence and Risk* (Dublin: Stationery Office, 1999); interview with former probation and welfare officer Julian Pugh.

76 *Report of the Steering Group on Prison Based Drug Treatment Services* (Dublin: Irish Prison Service, July 2000); interview with Mountjoy addiction specialist, Dr Des Crowley; interview with former probation and welfare officer Julian Pugh; interview with former Head of Nursing, Frances Nangle Connor; interview with former probation and welfare officer Tony Carlin.

77 Interview with anonymous former governor; interview with former prison Hospital Chief Officer, John Ramwell; Weston and Berridge, *HIV/AIDS in the 1980s and 1990s*; Duke, *Drugs, Prisons and Policy-Making*, 98.

78 *Report of the Director of Health Care for Prisoners, April 1992–March 1993* (London: HM Prison Service, 1993), 36–8; Interview with former part-time Prison Medical Officer Dr Pat Lush; interview with former Chief Inspector of Prisons, Lord David Ramsbotham, 27 February 2017; interview with former prison Hospital Chief Officer, John Ramwell.

79 Emma Bell, *Criminal Justice and Neoliberalism* (Basingstoke: Palgrave Macmillan, 2014), 5.

80 Interview with former Chief Inspector of Prisons, Lord David Ramsbotham.

81 Health Promotion Research Unit, LSHTM, *Disinfecting Tablets Pilot Project 1998: An Evaluation* (London, 1998); Weston and Berridge, *HIV/AIDS in the 1980s and 1990s*.

82 Duke, *Drugs, Prisons and Policy-Making;* Interview with former Chief Inspector of Prisons, Lord David Ramsbotham; Interview with Roger Howard, formerly of NACRO and SCODA.

83 Interview with former HMPS Prison Governor, Stephen Twinn; Weston and Berridge, *HIV/AIDS in the 1980s and 1990s*; HM Prison Service Order 3550, ‘Clinical Services for Substance Misusers’.

